# Determination of Selected Hydroxylated PAHs in Urine Samples of Individuals Consuming Grilled Marshmallows

**DOI:** 10.3390/molecules30183787

**Published:** 2025-09-18

**Authors:** Magdalena Szumska, Maciej Maciejczyk, Beata Janoszka, Aleksandra Damasiewicz-Bodzek, Agnieszka Nowak, Krystyna Tyrpień-Golder

**Affiliations:** Department of Chemistry, Faculty of Medical Sciences in Zabrze, Medical University of Silesia, 40-055 Katowice, Poland; mszumska@sum.edu.pl (M.S.); d201056@365.sum.edu.pl (M.M.); aleksandra.bodzek@sum.edu.pl (A.D.-B.); agnieszkanowak@sum.edu.pl (A.N.)

**Keywords:** grilling, marshmallows, PAHs, 1-hydroxypyrene, 9-hydroxyphenanthrene, chromatography, urine

## Abstract

Marshmallows are confectioneries that are popular among children and teenagers around the world. Barbecues and the consumption of grilled marshmallows, especially by children, have become fashionable in many countries. Grilled marshmallows may contain carcinogenic polycyclic aromatic hydrocarbons (PAHs). Hydroxy-PAHs (OH-PAHs) concentration in the urine of volunteers after the consumption of grilled marshmallows, as biomarkers of exposure to PAHs, have been determined. A total of 24 participants consumed marshmallows grilled under similar conditions. Urine samples were collected before and after the consumption of grilled marshmallows. 1-hydroxypyrene and 9-hydroxyphenanthrene concentrations in urine samples were determined using the HPLC-FLD technique after enzymatic hydrolysis and isolation by solid-phase extraction (SPE). The average concentration of 1-hydroxypyrene was 0.21 ± 0.16 µg/g creatinine and of 9-hydroxypenanthrene was 2.78 ± 2.55 µg/g creatinine. The concentrations of OH-PAHs in the urine of volunteers eating colored grilled marshmallows were higher compared to the consumption of white ones. In the case of 9-hydroxyphenanthrene this difference was statistically significant *p* < 0.05. Grilled marshmallows constitute a source of exposure to PAHs, especially in the group of children and adolescents. Even consumption of small or moderate amounts of grilled marshmallows resulted in a significant increase in concentrations of PAH metabolites in the urine compared to the level of these compounds before the intake.

## 1. Introduction

Marshmallows are confectionery products popular among children, teenagers, and adults all over the world. One of the fashionable ways to consume them is grilling over the bonfire or in the oven and even over a candle. Although they do not represent any beneficial nutritional value, marshmallows are generally safe for consumption. However, the grilling process may introduce certain changes in the product’s chemical composition, leading to the formation of potentially harmful compounds. Therefore, frequent consumption of grilled marshmallows may have adverse health effects and can constitute an additional source of exposure to dangerous compounds, including carcinogenic polycyclic aromatic hydrocarbons (PAHs) [1].

In general PAHs exhibit genotoxicity, mutagenicity, and carcinogenicity, and they can also affect reproduction. According to the International Agency for Research on Cancer (IARC), many of these compounds are possibly carcinogenic (Group 2B), probably carcinogenic (IARC Group 2A), or even carcinogenic to humans (e.g., BaP) (Group 1) [2]. It has been proven that chronic exposure to carcinogenic PAHs is associated with lung, stomach, bladder, skin, and breast cancer [3,4].

In the human body, PAHs undergo complex transformations, leading to the formation of reactive metabolites such as epoxides and phenols. PAHs are also metabolized into other compounds, including numerous quinones [5,6].

PAH metabolites, especially mono-hydroxy derivatives, may be excreted in the urine as free hydroxy-PAHs (OH-PAHs) and as glucuronide and/or sulfate conjugates. Mono-hydroxy derivatives are commonly used as biomarkers of human exposure to PAHs [7,8,9]. Native PAHs released into the atmosphere during the combustion of fuels undergo photooxidation and give rise to hydroxy-PAHs [10,11,12]. Airborne combustion products may therefore contain hydroxy-PAHs and enter the body. Exposure to OH-PAHs varies with the season, possibly due to photochemical reactions involving sulfur and nitrogen oxides [13]. Climate and lifestyle changes (including eating habits) are among the factors that can affect PAH exposure levels. For example, Canadians inhabiting subarctic northern Canada are exposed to higher levels of PAHs than the general Canadian population [14].

Hydroxy-PAHs, as metabolites of PAHs, are markers of exposure to these xenobiotics from various sources. Studies confirm that their determination in urine can be used to assess PAH exposure from the diet, too [15,16,17]. The PAH metabolite most commonly used in studying exposure to these compounds is 1-hydroxypyrene [8,18,19].

Since the relative content of pyrene compared to other PAHs is rather constant in environmental samples, and pyrene is a plentiful constituent of all PAH mixtures, 1-hydroxypyrene was and still is chosen for analysis and assessment of both occupational and environmental exposure to PAHs [7].

Among other hydroxy-PAHs, phenanthrene metabolites are another group that is often studied [8,20,21,22]. Although the level of hydroxy-phenanthrenes in urine is linked to occupational exposure, the hydroxy-metabolites of phenanthrene were reported to be sensitive markers related to diet PAH exposure, particularly from eating grilled or smoked food. It was observed that concentrations of hydroxy-phenanthrenes in urine samples were substantially higher than those resulting from smoking-related exposure [8].

In general, hydroxy–PAHs seem to show higher biological activity compared to their parent PAHs because they can interact and form adducts with DNA [23]. Recent research emphasizes the influence of PAH metabolites on the metastasis of liver cancer cells [24]. Higher blood pressure, higher heart rate, and atherosclerosis occur due to exposure to PAH metabolites [25]. The exposure to PAHs has also been linked to the onset of diabetes mellitus, and studies have shown a strong association between the urinary metabolite levels of PAHs and the risk of diabetes mellitus type 2 in the general population [26]. PAH exposure may even influence gut microbiota, which has a profound impact on human health, particularly for children, as the composition of gut bacteria is associated with the neurodevelopment of children. Study data suggest that this modification in gut microbiota development, especially during the second and third year of life, may be linked with neurodevelopment disorders in children [27]. The possibility of hydroxy-PAHs interacting with DNA and forming adducts can definitely lead to serious health problems in humans, especially in children, since their metabolism of PAHs is different from that in adults. Children seem more susceptible to PAHs and more vulnerable to PAHs exposure. A study conducted by Huang et al. revealed that urinary 1-hydroxypyrene concentration in children (6–11 years old) was about 30% higher compared to in adults under the same conditions. It indicates that children have higher potential health risks connected with PAHs exposure [21]. Moreover, some studies found association between PAHs exposure and risk of obesity, which was more apparent in children, females, and smokers [28]. Additionally, it was demonstrated that exposure to polycyclic aromatic hydrocarbons is among the causative factors of attention deficit hyperactivity disorder (ADHD) [29].

Even prenatal PAHs exposure may have a serious impact on the future health of a newborn. A growing body of toxicological research provides evidence of the neurotoxic effects of prenatal PAHs exposure. PAHs may harm the developing brain since they are able to transfer across the placenta, cross the blood–brain barrier, and accumulate in the brain [30,31]. The possible endocrine disrupting properties of these compounds and their metabolites may affect fetal airways and impair immune system development. It was observed that OH-PAHs metabolite concentrations in the urine of newborns were strongly associated with birth length, placental weight, and Apgar score at the fifth minute of life [32].

In presented study, we have focused mainly on diet-derived exposure to PAHs. We chose two hydroxy-PAH metabolites: 1-hydroxypyrene that can be feasibly identified at low levels and is reliable and well-established marker of exposure to PAHs; and one of the phenanthrene metabolites, namely 9-hydroxyphenantrene. The choice of phenanthrene metabolite was related to our previous findings concerning grilled marshmallows and their PAHs content. The concentration of phenanthrene determined in grilled marshmallows was the highest among all determined PAHs [1]. Moreover, a recent study indicated that these two particular hydroxy metabolites (1-hydroxypyrene and 9-hydroxyphenanthrene) can lead to the acceleration of DNA methylation, which is linked to human aging, a higher rate of age-related diseases, and epigenetic alterations [33].

The determination of hydroxy-PAHs present in biological and environmental samples at trace levels is most often performed using gas chromatography coupled with mass spectrometry (GC-MS) [34,35,36,37], liquid chromatography with mass spectrometry (LC-MS), or high-performance liquid chromatography (HPLC) in combination with fluorescence detection (HPLC-FLD) [8,16,37,38,39,40]. However, gas chromatography-based methods require the conversion of OH-PAHs into less polar derivatives, such as silyl compounds [35]. Therefore, the widely available, sensitive, and specific HPLC-FLD technique is one of the methods frequently used to measure OH-PAH concentrations in environmental and biological samples with different matrices [41,42,43,44].

The determination of OH-PAHs using any of the techniques mentioned above requires adequate preparation of the biological sample. Most often, such samples undergo enzymatic hydrolysis, followed by liquid or solid-phase extraction (SPE) [8,16,38,39,43,45].

Marshmallows are treats that children enjoy eating during barbecue parties or around campfires. It seems important to know whether eating grilled marshmallows constitutes an additional source of PAHs exposure for humans. Therefore, the aim of our study was to determine the levels of two selected PAH metabolites, namely 9-hydroxyphenanthrene and 1-hydroxypyrene, in urine samples collected from individuals before and after the consumption of grilled marshmallows. The study group consisted mainly of children and, to a lesser extent, young adults. The measurements were performed using high-performance liquid chromatography with fluorescence detection.

## 2. Results and Discussion

Food is one of the most important factors in human exposure to PAHs [46,47,48,49]. Unprocessed products have a relatively low content of PAHs, but thermal processing methods such as grilling, smoking, and drying contribute to the formation and accumulation of these harmful compounds in food [1,50,51]. PAHs that enter the body are metabolized into hydroxylated derivatives, which are conjugated to sulfates or glucuronides and subsequently excreted from the body. Bile, feces, urine, and milk are the principal elimination routes of PAH metabolites, however special attention has been given to urine since its collection is non-invasive, which is particularly important for children [3,40,52].

Previous studies have confirmed that grilled marshmallows contain polycyclic aromatic hydrocarbons in concentrations of several ng/g [1]. Therefore, we undertook further research to answer the question of whether an increase in the concentration of PAH metabolites in consumers’ urine can be detected after eating grilled marshmallows. Two metabolites were selected for the assessment of PAHs exposure: 1-hydroxypyrene and 9-hydroxyphenanthrene, since their parent PAH compounds were detected in high concentration in analyzed grilled material from our previous study [1], particularly high in the case of phenanthrene which was consistent with their metabolite concentrations in urine.

Studies performed by Motorykin et al. have demonstrated a positive association between ingested levels of phenanthrene and pyrene and the urinary levels of their metabolites, proving that metabolites of phenanthrene and pyrene can be reliable biomarkers of PAHs exposure [9].

**Figure 1:**
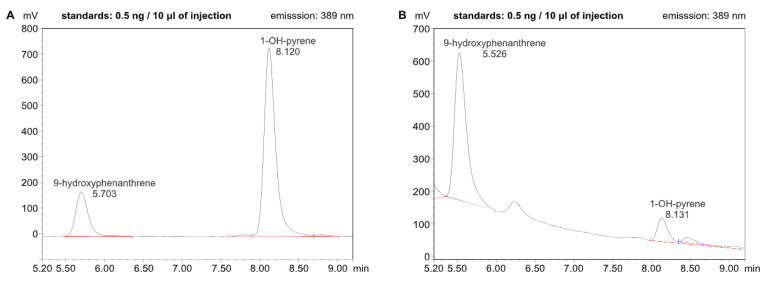
HPLC-FLD chromatograms obtained for (**A**) the standards 1-hydroxypyrene and 9-hydroxyphenanthrene and (**B**) for the urine sample of a volunteer consuming grilled marshmallows in which 1-hydroxypyrene and 9-hydroxyphenanthrene were identified.

In our study each urine sample of the volunteers was analyzed according to the procedure described in Methods (Section 3.7), including hydrolysis, SPE, and HPLC-FLD steps. The HPLC chromatogram obtained for the standards 1-hydroxypyrene and 9-hydroxyphenanthrene is shown in Figure 1A, and a chromatogram of the urine sample in which both mentioned monohydroxy PAH derivatives were identified, is shown in Figure 1B. The detection was performed using a fluorescence detector at excitation 242 nm and emission 389 nm wavelengths, respectively.

Validation parameters of the determination procedure of 1-hydroxypyrene and 9-hydroxyphenanthrene are presented in Table 1. The recovery rates of OH-PAHs were determined by applying analysis of unspiked and spiked urine samples at three concentration levels, different for each of the analyzed compounds. For 9-hydroxyphenathrene they were 30 ng/mL, 60 ng/mL, and 100 ng/mL, and for 1-hydroxypyrene 10 ng/mL, 30 ng/mL, and 60 ng/mL of urine. To calculate the recovery, the formula presented below was used:

$$\mathrm{recovery}\;=\;\frac{C_{1}\;-\;C_{2}}{C}\;\times \;100\%$$

where C_1_ is the concentration of OH-PAHs in the urine sample with added standards (ng/mL), C_2_ is the concentration of OH-PAHs in the urine sample (ng/mL), and C is the amount (ng) of standard added to 1 mL of urine sample. The recovery rates ranged from 61.3% (9-hydroxyphenanthrene, 100 ng/mL of spiked urine) to 80.1% (1-hydroxypyrene, 10 ng/mL of spiked urine). The mean recoveries of 1-hydroxypyrene and 9-hydroxyphenanthrene in urine samples were 71.8% and 67.8%, respectively. Our recovery rates are lower than those determined by Jongeneelen et al. for samples of urine spiked with 1-hydroxypyrene (83–88%) [41]. Importantly, the authors of the paper used SPE-C18 cartridges of different brands which may have affected the recovery rates. However, it is worth noting that Jongeneelen et al. obtained the highest recovery rates for the lowest spiking level (8.8 ng/mL), similarly to our study [41]. According to the literature data, when SPE-C18 cartridges are used for the extraction of PAHs from human excreta, recovery values range from 50% to over 80%. These values depend primarily on the brand of SPE cartridge [8]. High recovery rates (80–120%) are obtained when deuterated 1-hydroxypyrene-d9 standard is used [53]. The values obtained in our study (Table 1) are not among the highest when compared to the studies referenced in the paper, however they fit into the range suggested in the Official Journal of the European Communities concerning the performance of analytical methods and the interpretation of results (more than 50% for compounds analyzed at the level of ng/mL) [54].

The limits of detection (LOD) were calculated based on the signal-to-noise ratio S/N = 3 for 10 µL injection of standard solution onto the column. They were determined to be 0.15 ng/mL for both 9-hydroxyphenanthrene and 1-hydroxypyrene. The limit of quantification (LOQ) was calculated as 3 × LOD [55].

The lowest 9-hydroxyphenanthrene and 1-hydroxypyrene concentrations that could be determined in urine samples were 0.025 ng/mL of urine for the method of preparation, extraction, and HPLC-FLD determination described in Section 3.7.

The intra-day precision (repeatability) of the method was calculated as relative standard deviation (RSD%) of data obtained on the same day (n = 5) for samples with concentrations 10 ng/mL, 25 ng/mL, 50 ng/mL, and 100 ng/mL (for 10 µL injection). These ranged from 0.3% (1-hydroxypyrene, 25 ng/mL) to 3.0% (1-hydroxypyrene, 10 ng/mL). The inter-day precision (intermediate precision) of the method was also calculated as RSD% over five days, and it ranged from 0.9% (9-hydroxyphenanthrene, 10 ng/mL) to 5.6% (1-hydroxypyrene, 10 ng/mL). The HPLC-FLD method allowed us to determine selected hydroxy PAHs with sufficient recovery and satisfactory repeatability.

HPLC-FLD methods were often used by other authors for the determination of OH-PAHs in biological or environmental samples [41,42]; however, a different mobile phase was used for the chromatographic separation of hydroxylated PAHs isolated from airborne particulates [42] and sediments [43,44].

OH-PAH concentrations in urine samples from volunteers who consumed grilled marshmallows were normalized for changes in an individual’s urine flow rate by creatinine concentration (µg/g creatinine). The creatinine concentration in these samples ranged from 44 mg/dL to 243 mg/dL. The concentrations of 1-hydroxypyrene and 9-hydroxyphenanthrene in these urine samples per g of creatinine were approximately 0.21 ± 0.16 µg/g and 2.78 ± 2.55 µg/g creatinine, respectively.

**Figure 2:**
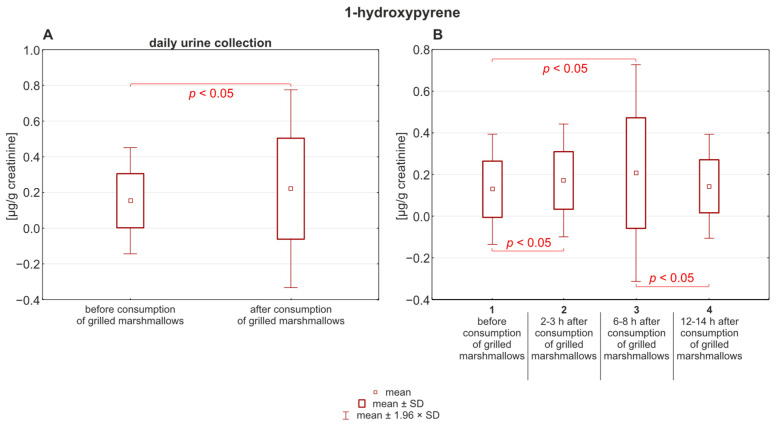
(**A**) Concentration of 1-hydroxypyrene in urine before and after the consumption of grilled marshmallows. (**B**) Distribution of 1-hydroxypyrene over time after consumption.

The results obtained from testing urine samples collected before and after the consumption of grilled marshmallows indicate that the differences between the mean concentrations of 1-hydroxypyrene before and after the consumption of grilled marshmallows were statistically significant *p* < 0.05 (Figure 2).

**Figure 3:**
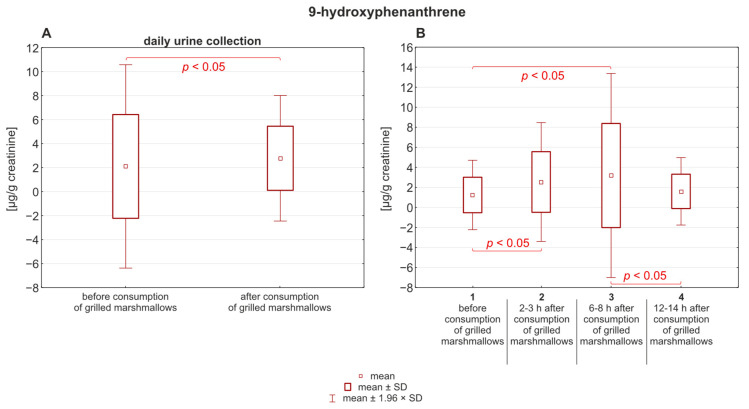
(**A**) Concentration of 9-hydroxyphenanthrene in urine before and after the consumption of grilled marshmallows. (**B**) Distribution of 1-hydroxyphenanthrene over time after consumption.

The same applies to 9-hydroxyphenanthrene (Figure 3), although in this case the pre-consumption exposure varied and showed greater diversity of concentrations than post-consumption level, compared to 1-hydroxypyrene.

The mean concentrations of the determined hydroxy-PAHs in the urine of volunteers consuming white and colored grilled marshmallows are presented in Table 2.

Similarly to our previous studies [1], in which we found higher PAH concentrations in grilled colored marshmallows than in white ones, the concentrations of both analyzed OH-PAHs were higher in the urine of volunteers consuming grilled colored marshmallows. However, a statistically significant difference between OH-PAH concentrations in the urine of people consuming white or colored marshmallows was only found for 9-hydroxyphenanthrene (*p* < 0.05). Its concentration in the tested samples was more than 10 times higher than that of 1-hydroxypyrene. We are aware that a certain limitation of this comparison is the small sample size.

Phenanthrene and pyrene are among the PAHs most frequently found in different environments and in various food matrices [1,40,56]. According to the International Agency for Research on Cancer (IARC), phenanthrene is not classified as a carcinogen for humans. However, according to the opinion of the Scientific Panel on Contaminants in the Food Chain of the European Food Safety Authority (EFSA), this compound and its hydroxyl derivatives may be present in food together with other more harmful, carcinogenic PAHs [34,57]. Moreover, the concentration of total phenanthrene metabolites was higher than pyrene concentration in breast milk samples in study performed by Oliveira et al. [40].

Most studies published to date have focused on the determination of 1-hydroxypyrene, as monohydroxylated PAH derivatives are most frequently the target of studies [8]. Jeng and Pan reviewed the current knowledge on urinary 1-hydroxypyrene as a biomarker of environmental and occupational exposure to polycyclic aromatic hydrocarbons (PAHs). They presented intra- and inter-individual variation in urinary 1-hydroxypyrene concentrations, the kinetics of 1-hydroxypyrene metabolism and excretion, and correlations between urinary 1-hydroxypyrene and parent PAH concentrations to investigate whether urinary 1-hydroxypyrene can serve as a reliable biomarker of PAHs exposure. They also presented evidence on PAH-related health effects and 1-hydroxypyrene associations in humans to determine the potential applications of urinary 1-hydroxypyrene in predicting multiple diseases associated with PAHs exposure [58].

Urine is the primary route of excretion of PAHs and their metabolites. Analyzing urinary 1-hydroxypyrene in pregnant women in Rochester, Lin et al. found that changes in concentration of this compound were related to household income, education level and distance of residence from means of transport that were the source of PAH emissions [59]. For the health of mothers and their newborn children, Portuguese scientists determined the presence of PAHs and their six selected metabolites (OH-PAHs) in the milk of breastfeeding women [40]. The paper highlights the importance of analyzing PAHs and their main metabolites in future biomonitoring studies during breastfeeding to better assess potential health risks for mothers and their breastfed infants.

In general, children and especially infants are extremely susceptible to adverse health consequences after exposure to toxic compounds like PAHs. Their metabolic and physiological defense functions are not fully developed. Enzymes such as cytochrome P450 taking part in the detoxification of xenobiotics are less efficacious in early life periods compared to adult life. Therefore, special attention to the health of children exposed to toxic substances is crucial [18]. Concentrations of PAH metabolites tend to be higher in younger children compared to older ones, and higher in children and adolescents compared to non-smoking adults [21,60]. In the study conducted by Fernández et al., 11 PAH metabolites including 1-hydroxypyrene and 9-hydroxyphenethrene were analyzed in a group of children in the Valencian Region (Spain). The mean concentrations of 1-hydroxypyrene and 9-hydroxyphenethrene were lower than our mean results before the consumption of marshmallows. Huang et al., who conducted a largescale meta-analysis of PAHs exposure in a group of children and adolescents from Europe, Asia, and North America, noted a much higher 1-hydroxypyrene concentration compared to our results from before the consumption of marshmallows [21]. Results higher than ours were also reported by Trask et al., who analyzed blood and urine samples obtained from children and adolescents living in rural Bangladesh [20]. In the study of Pérez-Maldonado et al., the factor of habitation area was included in the measurement of PAHs metabolite 1-hydroxypyrene. The authors divided the living areas into five groups: urban areas with low vehicular traffic, urban areas with heavy vehicular traffic, rural areas near to a big municipal landfill, rural areas with a brick kiln industry, and rural areas using wood combustion. The highest levels of analyzed OH-metabolite were noted for the brick kiln industry area and the lowest for the urban area with low vehicular traffic. The groups living close to big municipal landfills and urban areas with heavy vehicular traffic were characterized by 1-hydroxypyrene level similar to ours. Indeed, our study participants live in areas with moderate traffic [18]. A study carried out in Germany showed urine levels of 1-hydroxypyrene closer to ours (0.087 μg/g creatinine vs. 0.18 µg/g creatinine before eating grilled marshmallows), but 9-hydroxyphenathrene levels were lower than in our study group [61]. These differences between countries and regions reflect the influence of diet habits and environmental pollution.

In our study, we did not analyze boys and girls separately due to the small investigation group. Fernández et al. noted that girls presented higher 9-hydroxyphenanthrene and 1-hydroxypyrene metabolites levels compared to boys of the same age group. Huang et al. noticed a similar tendency; however, the significance of the results seemed to depend on different statistical methods applied [21,60]. Girls at age 15 exhibited consistent positive associations between OH-PAH (including 1-hydroxypyrene) concentration and cholesterol, triglyceride, and CRP ratios as well as with overall cardiometabolic risk scores in the study conducted by Trask et al. [20].

When it comes to the distribution of PAHs from diet, data obtained by Lapole et al. in an animal study suggests that the highest pyrene concentration in plasma was found within the first 2 h after exposition to PAHs, and it fell rapidly. In case of phenanthrene, its concentration increased slightly during the first hour after oral intake and slowly decreased until 20 h. The excretion of its metabolites via urine was the highest after 8 h; however, they also were detected at significant levels during the period of 0 to 4 h [62].

Studies using deuterium-labeled polycyclic aromatic hydrocarbons (PAHs), including phenanthrene and pyrene, administered orally in a single dose (0.02–0.04 mg/kg) to eight healthy adults showed that monohydroxylated PAHs were excreted in urine with half-life values ranging from 3.4 to 11.0 h [4]. In our study, the mean concentrations of urinary 1-hydroxypyrene and 9-hydroxyphenanthrene (as shown in Figure 2 and Figure 3) were the highest 6 to 8 h after the grilled marshmallows were eaten (0.21 ± 0.28 µg/g creatinine for 1-hydroxypyrene; 2.47 ± 2.50 µg/g creatinine for 9-hydroxyphenanthrene). The greatest variability (highest SD) was also recorded during this time period. It is likely that individual differences in the rate of xenobiotic metabolism are most evident during this time period. However, the highest median value for 1-hydroxypyrene was observed after 2–3 h, which was 0.17 ± 0.21 µg/g creatinine. In the case of 9-hydroxyphenanthrene, the highest median value (3.49 ± 8.65 µg/g creatinine) was observed 6–8 h after the grilled marshmallows were eaten. According to Motorykin et al., the median half-lives for the parent PAHs are 3.3 h for pyrene and 2.2 h for phenanthrene. The shorter elimination time for phenanthrene may explain the higher concentration of 9-hydroxyphenanthrene in urine compared to 1-hydroxypyrene [9]. In our study the mean concentrations of the analyzed hydroxy-PAHs decreased almost to the initial state after 24 h. For 1-hydroxypyrene it changed from 0.19 ± 0.20 µg/g creatinine to 0.17 ± 0.19 µg/g creatinine. In the case of 9-hydroxyphenanthrene, that change was more noticeable: from 3.6 ± 8.53 µg/g creatinine to 1.91 ± 2.19 µg/g creatinine. The concentration of 1-hydroxypyrene in almost every sample was above the limit of quantification. However, in four samples the initial concentration of 9-hydroxyphenanthrene before the consumption of grilled marshmallows was below the limit of quantification, and in five samples it decreased below the LOQ in the last micturition.

**Figure 4:**
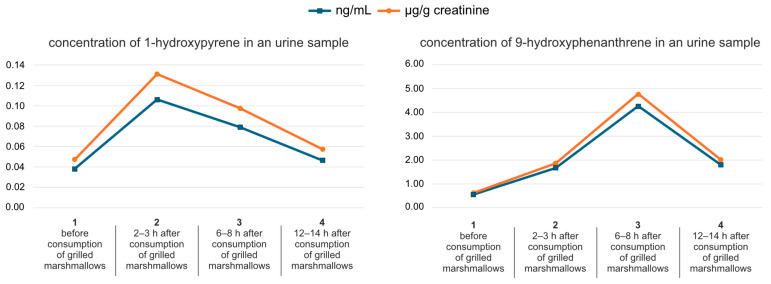
Examples of different concentration distributions of hydroxy-PAHs determined in urine samples collected before (sample collection no. 1) and after the consumption of grilled marshmallows (sample collection no. 2, 3, and 4).

Examples of daily distributions of hydroxyl-PAHs concentrations determined in urine samples of volunteers consuming grilled marshmallows are presented in Figure 4.

**Figure 5:**
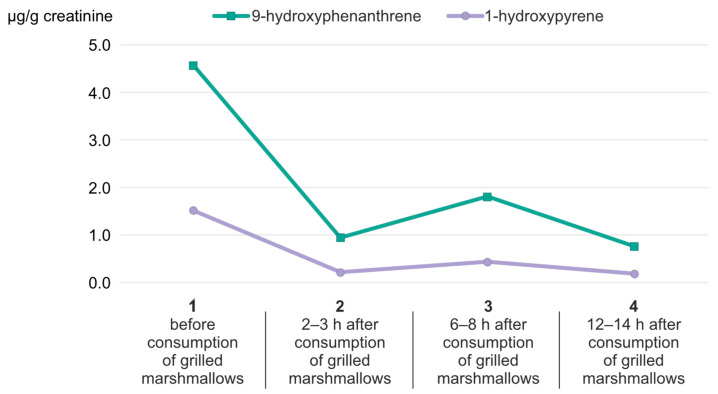
Distribution of hydroxy-PAH concentrations determined in urine samples collected from a person who grilled and then consumed white grilled marshmallows.

The concentration of OH-PAHs in urine might depend on the volume of drank beverages, the frequency of urination, as well as on the exposure to other sources of PAHs (place of residence related to air quality and food consumed) [3,52]. Moreover, the highest concentration of determined OH-PAHs was often observed in the first or the second urine sampling after consuming the marshmallows (Figure 4), which definitely depends on the individual rate of xenobiotic metabolism [7,39,52]. It is also worth noting that in the urine samples of the person who grilled the marshmallows, the initial concentrations of both tested compounds were high just after grilling and before the consumption of marshmallows (Figure 5) [7,39,52]. Studies confirm higher exposure to PAHs in people standing close to the grill and exposed to barbecue fumes. Not only the oral route, but also inhalation and dermal absorption have to be taken into consideration in such cases [63,64]. Inhalation exposure appears to be of particular importance. The respiratory system is characterized by an exceptionally large absorptive surface that enables rapid diffusion of gases, vapors, and small particles directly into the bloodstream [65]. The inhalation route bypasses the body’s key hepatic detoxification mechanisms. Although the lungs contain metabolic enzymes (including cytochrome P450), their activity is much lower than in the liver. Thus, almost the full active dose of the inhaled substance reaches the systemic circulation [66,67]. Furthermore, some inhaled substances can reach the brain directly via the nasal–cerebral pathways, bypassing the blood–brain barrier. Transneuronal transport through the olfactory and trigeminal nerves constitutes an extremely dangerous route of exposure for neurotoxic xenobiotics [68,69]. Frequent inhalation exposure to PAHs may also pose a health risk to the lungs themselves [70]. The exposure to PAHs during grilling also depends significantly on the type of grill and fuel. The risk factors associated with such exposure in children are also higher than in adults [71].

Few studies have been published on the excretion rates of parent PAHs and OH-PAHs following oral exposure. In study by Motorykin at al., the metabolism and excretion rates of four parent PAHs and ten OH-PAHs after consuming smoked salmon were examined. The participants of the study consumed 50 g of traditionally smoked salmon with breakfast, and five urine samples were collected over the 24 h. OH-PAH concentrations initially increased. The concentrations of most parent PAHs and their metabolites returned to background levels 24 h after ingestion which was also observed in our study [9].

The concentrations of metabolites excreted in urine undoubtedly depend on the type and the amount of food consumed. According to Li et al., the ingested barbecued chicken mass correlated with concentrations of 3- and 4-hydroxyphenanthrenes. Moreover, the various routes of exposure also influenced the half-life of investigated metabolites (from 4.4 to 29 h for metabolites of phenanthrene and 4.4 to 12 h for pyrene metabolites). The median concentration of 1-hydroxypyrene was higher than in our study (1.86 µg/g creatinine), which also confirms the influence of the type of food consumed. The observed OH-PAH levels reached maximum in less than 8.5 h among the participants who were eating grilled chicken, and the majority of the urinary OH-PAHs were excreted within 12 h after the exposure [17].

Environmental factors were believed to be contributors to human aging and human health status. PAHs are ubiquitous in the environment, and humans are exposed to PAHs at background concentrations, particularly in more industrialized regions and larger metropolitan areas such as Upper Silesia in Poland; nevertheless, the need for dietary control in biomonitoring PAHs exposure is undoubtedly necessary [72].

Reducing exposure to PAHs through the diet in the general population, especially vulnerable groups such as children, can be made by establishing appropriate regulations and biomonitoring [49].

## 3. Materials and Methods

### 3.1. Chemicals and Reagents

Sodium azide (analytical-reagent grade) for preserving urine samples, glacial acetic acid (analytical-reagent grade) to prepare acetate buffer, acetone (analytical-reagent grade), methanol (HPLC-grade), and natrium hydroxide (analytical-reagent grade) were from Avantor™ Performance Materials (Gliwice, Poland). Hydrochloric acid (37%, analytical grade) was delivered from Chemland (Katowice, Poland). Water was obtained from a water deionizer system Polwater DL-2 (Labopol-Polwater, Kraków, Poland). *Helix pomatia* (Merck, Darmstadt, Germany) mixture of β-glucuronidase/arylsulfatase enzymes was used for enzymatic hydrolysis.

### 3.2. Hydroxy-PAHs Standards

**Figure 6:**
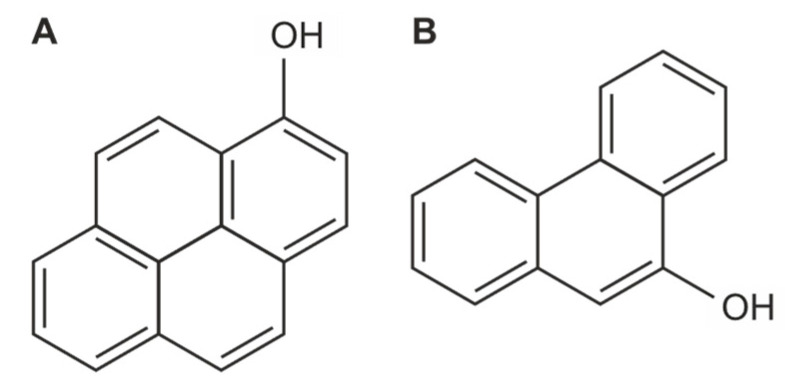
Structure of 1-hydroxypyrene (**A**) and 9-hydroxyphenanthrene (**B**).

1-hydroxypyrene (purity > 98%) and 9-hydroxyphenanthrene (purity > 98%) were purchased from Sigma Aldrich (St. Luis, MO, USA). The structures of these compounds are shown in Figure 6. Standard stock 0.1 mg/mL solutions of OH-PAHs were used to prepare 1 μg/mL standard mixtures in methanol. The standard mixture was diluted to prepare solutions at concentrations ranging from 0.5 ng/mL to 250 ng/mL. Detection limits, quantification limits, and standard calibration curves were prepared with use of the diluted standard mixtures.

### 3.3. Apparatus

Solid-phase extraction (SPE) system (J.T. Baker, Gross Gerau, Germany) and SPE columns J.T.Baker^®^ BAKERBOND™ SPE octadecyl (C18), 500 mg/6 mL (Avantor VWR International Sp. z o.o., Gliwice, Poland) were applied to purify the hydroxy PAHs fraction. An evaporation system under nitrogen stream (VLM GmbH, Bielefeld, Germany) was used for solvent evaporation.

OH-PAHs determination was conducted using a high-performance liquid chromatography (HPLC) system Ultimate 3000 TSL (Dionex Softron, Germering, Germany), consisting of a thermostated autosampler WPS-3000 TSL (Dionex Softron, Germering, Germany), a column compartment TCC-3200 (Dionex Softron, Germering, Germany) with a Hypersil Green PAH column (particle size 5 µm; 250 × 4.6 mm I.D; Thermo Scientific, Waltham, MA, USA), and a fluorescence detector (FLD) (Shimadzu RF-2000, Kyoto, Japan). Analysis monitoring and data collection were carried out using Chromeleon software (version 6.80 SP2 Build 2284; Dionex Softron, Germering, Germany).

### 3.4. Grilled Marshmallows

The marshmallow grilling procedure was described in detail in our previous manuscript [1]. The marshmallows were grilled over a beechwood fire, lit, and maintained without artificial kindling. All were grilled in the same manner: four pieces at a time, on a grill skewer, at the same height above the fire for the same amount of time (average of about 30 s per 12 g), until they increased in volume and changed color (from caramel to brown).

### 3.5. Characteristics of Participants Consuming Grilled Marshmallows

The study involved 24 people aged from 8 to 18 years, 12 boys and 12 girls. The average age of the study participants was 12.6 years. The volunteers who consumed white (16) and colored (8) marshmallows mainly lived in the Podkarpackie and Silesian provinces of Poland. They were not exposed to polycyclic aromatic hydrocarbons through ingestion or inhalation more than the other residents of these regions. The study was approved by the bioethics committee—approval number PCN/CBN/0052/KB1/18/22, dated 12 April 2022. Participants in the experiment were approximately 50 m away from the marshmallow grilling site (except for the griller). The experiment was conducted on a sunny, warm summer day, away from an urbanized area. In addition, participants were asked not to eat smoked, grilled, or roasted foods the day before the experiment. These restrictions were intended to limit the study participants’ exposure to PAHs from sources other than grilled marshmallows.

### 3.6. Urine Sampling

All urine samples were collected from individuals consuming marshmallows during the same barbecue. After obtaining informed consent from the study participants or their parents, urine samples were collected before the consumption and after the consumption of grilled marshmallows. Urine samples from people just before eating grilled marshmallows were used as a control. Next, approximately 100 mL of urine was collected from each micturition, starting with the micturition about 2–3 h after consuming the marshmallows, then the sampling was made 6–8 and 12–14 h after the consumption. The last sampling was taken approximately within 24 h after consuming the marshmallows. A 24 h urine collection was not performed due to technical and logistical difficulties in its execution.

Creatinine in urine was determined spectrophotometrically as picrate according to the Jaffé method [73] by using the automated biochemical test Creatinine2 Reagent Kit 04T91 and an analytical system ABBOTT ALINITY C (Abbott Ireland Diagnostics Division Lisnamuck, Longford Co., Longford, Ireland). The measurements were performed based on the guidelines EP05-A3.25 [74].

### 3.7. Analysis of Hydroxy-PAHs in Urine

The analysis of selected hydroxy-PAHs was performed according to the sensitive method recommended by Jongeneelen et al. The pretreatment of samples was based on a scheme involving stages of enzymatic hydrolysis and SPE clean-up. The determination of metabolites was carried out according to our own modified method [41]. To achieve the repeatability of the analysis conditions and reduce the influence of concentration and pH fluctuation, all the required solutions (acetate buffer, eluents for HPLC analysis) were prepared before the beginning of research. Hydrolysis of the urine samples, OH-PAHs isolation, and HPLC-FLD analysis were conducted in repeatable, identical conditions.

#### 3.7.1. Hydrolysis

In order to release hydroxy-PAHs from the glucuronic and sulfuric acid complex, urine samples were treated with enzymes β-glucuronidase/arylsulfatase. First, 0.1 mol/L of hydrochloric acid was added to each 10 mL urine sample to obtain solutions of pH 5.5, followed by adding 10 mg of sodium azide. To the samples prepared in this way, 40 µL of enzyme (β-glucuronidase /arylsulfatase) and 2 mL of acetate buffer of 1 mol/L, pH = 4 were added. Next, the samples were incubated for 16 h at 37 °C, and after incubation the OH-PAHs were extracted using the SPE technique.

#### 3.7.2. Solid-Phase Extraction of the Hydroxy-PAHs-Containing Fraction

Extraction was carried out using C-18 500 mg/6 mL columns. Before applying the samples, the columns were conditioned by washing with 5 mL of methanol and 10 mL of deionized water. After applying the hydrolyzed urine samples, the columns were washed with 10 mL of deionized water and then dried. The analyzed compounds retained on the SPE bed were eluted with 5 mL of a mixture of methanol–acetone (1:1, *v*/*v*). The obtained solution was evaporated to dryness under a stream of nitrogen and then dissolved in 500 µL of methanol. The analysis was performed with the use of the HPLC-FLD technique.

#### 3.7.3. Determination of Hydroxy-PAHs by HPLC-FLD

High-performance liquid chromatography with fluorescence detection was used to determine the concentrations of selected hydroxy-PAHs in the urine sample extracts. The injection volume of each extract was 10 µL. The chromatographic separations on the Hypersil Green PAH column were performed under gradient conditions by using methanol and water. The eluent flow rate was as follows: 80% MeOH and 20% water from 0.0 to 3.0 min, then increasing to 100% MeOH until 5.0 min, followed by a plateau (100% methanol) until 10.0 min. From 10.01 min to 13.0 min, the MeOH content decreased to 80%. The flow of eluents of this composition was maintained until 17.0 min of analysis. The flow rate of the mobile phase was 0.8 mL/min. The chromatographic column temperature was set to 40 °C. Under these separation conditions, retention times were as follows: 5.67 min for 9-hydroxyphenatrene and 8.1 min for 1-hydroxypyrene. The fluorescence detection was performed by the following excitation and emission (Ex/Em) wavelengths: 242/389 nm. These wavelengths were selected based on the literature data [13,41].

Retention times were recorded for 9-hydroxyphenathrene and 1-hydroxypyrene standards. These values were compared with the retention times of compounds identified in extracts of urine samples in order to confirm the presence of OH-PAHs.

Quantitative analysis of OH-PAHs was performed using the injection of 10 µL of standard mixture (in MeOH), on the basis of linear calibration plots recorded in the range of 0.5 ng/mL to 250 ng/mL for 9-hydroxyphenanthrene and 0.5 ng/mL to 100 ng/mL for 1-hydroxypyrene. Calibration graphs were constructed using the least-squares method for eight points of concentration. The calibration coefficients *r* for the curves were above 0.999.

### 3.8. Statistical Analysis

Statistical analysis was performed using the Statistica 13.3 software (TIBCO Software Inc., Palo Alto, CA, USA). The results were compiled using basic parameters of descriptive statistics. The compatibility of the variable with normal distribution was checked by Shapiro–Wilk test. The Wilcoxon signed-rank test was used to compare the concentration of both hydroxy-PAHs in urine before and after grilled marshmallow consumption. The Mann–Whitney test was used to compare the mean urine PAHs concentration between the group consuming white and colored marshmallows. A *p* value < 0.05 was considered statistically significant.

## 4. Conclusions

The elaborated method of analysis of hydroxy-PAHs in urine samples proved to be an efficient analytical tool for the determination of biomarkers of PAHs exposure from grilled food. Our preliminary study indicated that even the consumption of small or moderate amounts of grilled marshmallows, white and colored, resulted in an increase in concentrations of analyzed 1-hydroxypyrene and 9-hydroxyphenanthrene, compared to the level of these compounds before the consumption. However, to elucidate the exact rates of PAHs exposure from grilled marshmallows, large-scale, long-term studies in conjunction with mechanistic studies are needed. Such studies would have to consider in more detail the effects of factors such as diet and environmental exposure, and assess dose–response relationships.

The lack of knowledge regarding carcinogenic substance content in food constitutes a social problem, especially among young people who are beginning the process of developing eating habits for future health status. Grilled confectionery products can be a new, additional source of exposure to PAHs, which should be taken into consideration, primarily in children and the adolescent population.

## Figures and Tables

**Table 1:** Basic parameters for validation of the OH-PAH determination method.

Parameter	9-Hydroxyphenanthrene	1-Hydroxypyrene
Calibration curve equation ^1^	y = 70.729x − 0.8354	y = 261.03x − 0.6396
Correlation coefficient [*r*]	0.9999	0.9996
LOD and LOQ [ng/mL] ^2^	0.15 and 0.50	0.15 and 0.50
LOQ [ng/mL urine]	0.025	0.025
Recovery % and (RSD%) ^3^ for the spiking levels:		
10 ng/mL urine	n.d. ^4^	80.0 (5.1)
30 ng/mL urine	72.1 (4.1)	73.2 (3.7)
60 ng/mL urine	69.9 (2.5)	62.1 (1.0)
100 ng/mL urine	61.3 (2.5)	n.d. ^4^
Intra-day and inter-day precision expressed as RSD% ^3^ for the concentration levels:		
10 ng/mL	1.3 and 0.9	3.0 and 5.6
25 ng/mL	1.0 and 1.3	0.3 and 4.3
50 ng/mL	0.5 and 1.2	1.1 and 4.1
100 ng/mL	0.6 and 1.2	0.4 and 1.8

^1^ Calibration curve range: 9-hydroxyphenanthrene—from 0.5 ng/mL to 250 ng/mL; 1-hydroxypyrene—from 0.5 ng/mL to 100 ng/mL (for 10 µL injection volume). ^2^ For 10 µL injection volume. ^3^ Number of analysis repetitions n = 5. ^4^ Not determined.

**Table 2:** Comparison of hydroxy-PAHs concentrations in urine samples from individuals who consumed grilled marshmallows without and with the division regarding type of marshmallows (colored and white).

	Consumption of Grilled Marshmallows		
Compound	Overall Consumption White and Colored (n = 24)	Only Colored (n = 8)	Only White (n = 16)
1-hydroxypyrene mean ± SD [µg/g creatinine]	0.21 ± 0.16	0.24 ± 0.20	0.19 ± 0.09
9-hydroxyphenanthrene mean ± SD [µg/g creatinine]	2.78 ± 2.55	3.07 ± 2.40 ^1^	2.65 ± 2.30 ^1^

^1^ *p* < 0.05 colored vs. white.

## Data Availability

The raw data supporting the conclusions of this article will be made available by the authors on request.
